# Dynamics of SARS-CoV-2 infection hospitalisation and infection fatality ratios over 23 months in England

**DOI:** 10.1371/journal.pbio.3002118

**Published:** 2023-05-25

**Authors:** Oliver Eales, David Haw, Haowei Wang, Christina Atchison, Deborah Ashby, Graham S. Cooke, Wendy Barclay, Helen Ward, Ara Darzi, Christl A. Donnelly, Marc Chadeau-Hyam, Paul Elliott, Steven Riley

**Affiliations:** 1 School of Public Health, Imperial College London, London, United Kingdom; 2 MRC Centre for Global infectious Disease Analysis and Abdul Latif Jameel Institute for Disease and Emergency Analytics, Imperial College London, London, United Kingdom; 3 Department of Infectious Disease, Imperial College London, London, United Kingdom; 4 Imperial College Healthcare NHS Trust, London, United Kingdom; 5 National Institute for Health Research Imperial Biomedical Research Centre, London, United Kingdom; 6 Institute of Global Health Innovation, Imperial College London, London, United Kingdom; 7 Department of Statistics, University of Oxford, Oxford, United Kingdom; 8 MRC Centre for Environment and Health, School of Public Health, Imperial College London, London, United Kingdom; 9 Health Data Research (HDR) UK London at Imperial College, London, United Kingdom; 10 UK Dementia Research Institute at Imperial College, London, United Kingdom; McMaster University, CANADA

## Abstract

The relationship between prevalence of infection and severe outcomes such as hospitalisation and death changed over the course of the COVID-19 pandemic. Reliable estimates of the infection fatality ratio (IFR) and infection hospitalisation ratio (IHR) along with the time-delay between infection and hospitalisation/death can inform forecasts of the numbers/timing of severe outcomes and allow healthcare services to better prepare for periods of increased demand. The REal-time Assessment of Community Transmission-1 (REACT-1) study estimated swab positivity for Severe Acute Respiratory Syndrome Coronavirus 2 (SARS-CoV-2) infection in England approximately monthly from May 2020 to March 2022. Here, we analyse the changing relationship between prevalence of swab positivity and the IFR and IHR over this period in England, using publicly available data for the daily number of deaths and hospitalisations, REACT-1 swab positivity data, time-delay models, and Bayesian P-spline models. We analyse data for all age groups together, as well as in 2 subgroups: those aged 65 and over and those aged 64 and under. Additionally, we analysed the relationship between swab positivity and daily case numbers to estimate the case ascertainment rate of England’s mass testing programme. During 2020, we estimated the IFR to be 0.67% and the IHR to be 2.6%. By late 2021/early 2022, the IFR and IHR had both decreased to 0.097% and 0.76%, respectively. The average case ascertainment rate over the entire duration of the study was estimated to be 36.1%, but there was some significant variation in continuous estimates of the case ascertainment rate. Continuous estimates of the IFR and IHR of the virus were observed to increase during the periods of Alpha and Delta’s emergence. During periods of vaccination rollout, and the emergence of the Omicron variant, the IFR and IHR decreased. During 2020, we estimated a time-lag of 19 days between hospitalisation and swab positivity, and 26 days between deaths and swab positivity. By late 2021/early 2022, these time-lags had decreased to 7 days for hospitalisations and 18 days for deaths. Even though many populations have high levels of immunity to SARS-CoV-2 from vaccination and natural infection, waning of immunity and variant emergence will continue to be an upwards pressure on the IHR and IFR. As investments in community surveillance of SARS-CoV-2 infection are scaled back, alternative methods are required to accurately track the ever-changing relationship between infection, hospitalisation, and death and hence provide vital information for healthcare provision and utilisation.

## Abstract 2

This study investigates how the severity of effects of the SARS-CoV-2 virus in England was affected by the mass vaccination campaign and emergence of Alpha, Delta and Omicron variants between May 2020 and March 2022.

## Introduction

Since its first detection in late 2019, Severe Acute Respiratory Syndrome Coronavirus 2 (SARS-CoV-2) has led to high levels of morbidity and mortality worldwide [1,2]. In England in late 2020, following the emergence of the Alpha variant, which has been linked to higher levels of transmissibility [3,4] and severity [5] (relative to wild-type variants), there was a rapid rise in infections leading to a surge in hospitalisations and deaths [6] and to intense pressure on the National Health Service (NHS). In order to stem the tide of infections, a national lockdown was introduced on 6 January 2021 [7], aimed at drastically reducing social contacts. Simultaneously, as this lockdown began, England began implementing a mass vaccination campaign and has since reached high levels of vaccine coverage [6]. Studies have found the vaccines to be highly effective against severe outcomes at the individual level [8] and have also been linked to reduced levels of transmission [9]. The combined effect of both lockdown and vaccinations led to a sharp decrease in cases, hospitalisations, and deaths during the first few months of 2021 [6].

After March 2021, lockdown restrictions were slowly lifted with phased reopenings [10]. Combined with the emergence of the Delta variant in England in April 2021, which has been linked to even greater levels of transmissibility than prior variants [11,12] and reduced vaccine effectiveness [13], the pandemic once more entered a phase of growth leading to high prevalence [14]. The last easing on 19 July 2021 removed all domestic legal restrictions and saw society reopen to an extent not seen since March 2020 [15]. Restrictions have not since been implemented at such a large scale in England, despite high prevalence levels during the summer and autumn of 2021 [16] and 2 large waves of infection [17] following the emergence of the Omicron variant and its BA.1 and BA.2 sublineages [16]. In deciding to implement or remove restrictions, the UK government made their main criteria that “Infection rates do not risk a surge in hospitalisations which would put unsustainable pressure on the NHS” [10]. Assessing trends between levels of infection and hospitalisation rates is therefore crucial in better informing governments and public health bodies so that restrictions can be appropriate and proportionate.

The infection fatality ratio (IFR) and infection hospitalisation ratio (IHR) measure the proportion of deaths and hospitalisations, respectively, among infected individuals. When they are known accurately, short-term forecasts of severe outcomes can be made using current estimates of infection levels and the estimated time-delay to severe outcomes [18,19]. Models forecasting future levels of infection [20–22] can also then easily be converted into forecasts of severe outcomes. Such forecasts can allow healthcare services to better prepare for periods of increased demand. Accurate estimates of the IFR and IHR have been made during the pandemic [23,24]. However, with the introduction of new variants, the rollout of vaccinations, the waning of vaccine effectiveness [25–27], and the effect of vaccine booster doses [28], the values of IFR and IHR can rapidly change.

The REal-time Assessment of Community Transmission-1 (REACT-1) study involved cross-sectional surveys over 19 rounds that aimed to test a random sample of the population of England for the presence of the SARS-CoV-2 virus [29]. Each round of the study occurred approximately monthly between May 2020 and March 2022, with between 95,000 and 175,000 individuals aged 5+ years taking part at each round. The study allowed the progression of the pandemic in England to be accurately measured [30] without the biases of routine reporting or other nonrandom sampling methods [31]. By accurately characterising the relationship between severe outcomes and these gold-standard data, real-time changes in the severity of infection with the virus can be detected and quantified with only a small delay (due to the time between infection and an occurrence of a severe outcome). We present here the relationship between the prevalence of infection estimated from round 1 to round 19 of REACT-1 (May 2020 to April 2022), and the daily number of deaths and hospital admissions in England over the same period, using the relationships to quantify the IFR and IHR of the pandemic in England. We further apply the methodology developed to the daily number of cases allowing us to estimate the case ascertainment rate (the proportion of infections identified with a positive test) of England’s mass testing programme.

## Results

### Quantifying the relationship between swab positivity and severe outcomes

**Fig 1:**
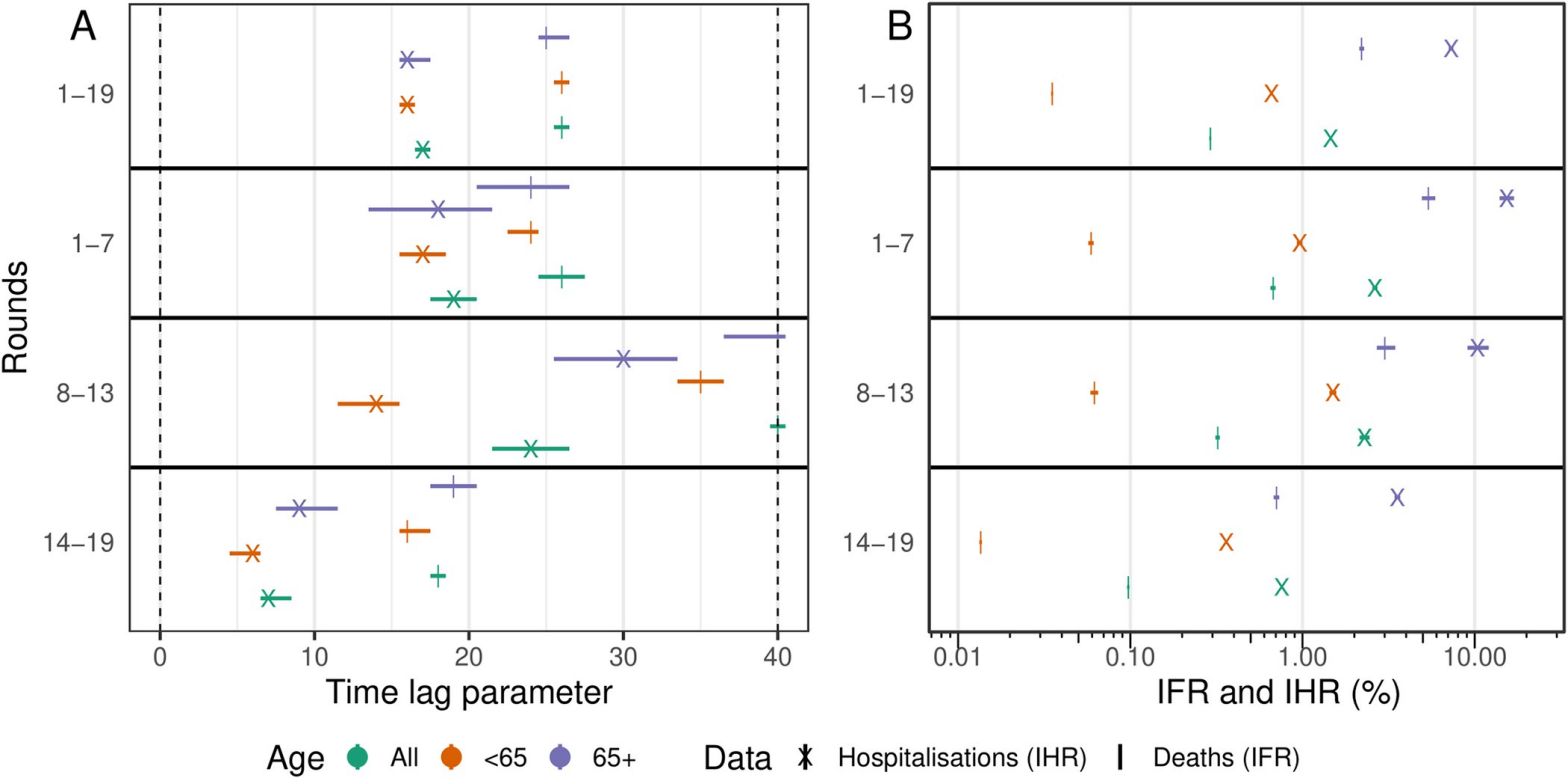
Comparison of parameters obtained by fitting to different subsets of the REACT-1 data. **(A)** Best-fitting time-lag parameter and 95% credible intervals for models fit to deaths (cross, time-lag between swab positivity and deaths) and hospitalisations (vertical line, time-lag between swab positivity and hospitalisations), for models fit to all age-groups (green), those aged 64 years and under (orange), and those aged 65 years and over (purple). **(B)** Best-fitting scaling parameter and 95% credible intervals on a *log*_10_ x-axis for models fit to deaths (cross) and hospitalisations (vertical line), for models fit to all age-groups (green), those aged 64 years and under (orange), and those aged 65 years and over (purple). All numerical values are provided in S1 Table.

**Fig 2:**
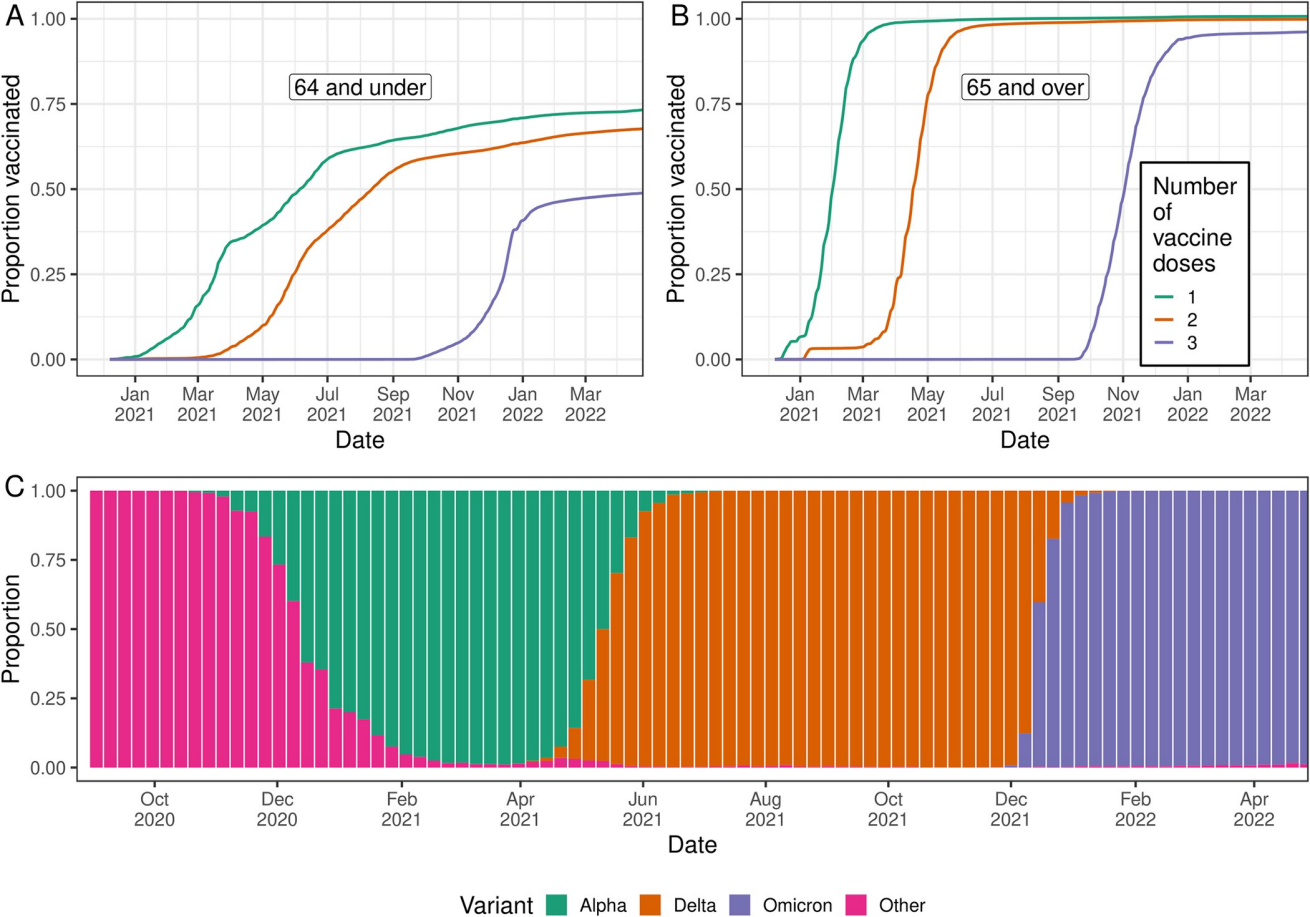
Vaccination coverage and variants responsible for infections in England, as inferred from public data sets. **(A, B)** Proportion of individuals who have been vaccinated with at least a single dose (green), proportion of individuals who have been vaccinated with at least 2 doses (orange), and the proportion of individuals who have received a third “booster” dose (purple), for those aged 64 years and under (A) and those aged 65 years and over (B). **(C)** Proportion of infections in England, for which lineages were detected, which were identified as the Omicron variant (purple), the Delta variant (orange), the Alpha variant (green), and any other lineage (pink). Data supporting this figure can be found in S1 Data.

The time-lag between swab positivity and severe outcomes decreased over the duration of the study. Fitting the time-delay model (see Methods) to rounds 1 to 7 (1 May to 3 December 2020) of REACT-1, we estimated a discrete time-lag of 19 (18, 20) days to hospitalisations, and a time-lag of 26 (25, 27) days to deaths (Fig 1, S1 Table). These estimates are in line with those obtained by fitting the same model to all 19 rounds of the data. Fitting the model to rounds 14 to 19 (9 September 2021 to 31 March 2022) of the study, we estimated much shorter time-lags of 7 (7, 8) days to hospitalisations and 18 (18, 18) days to deaths. Models fit to subsets of the data by age group (64 years and under, 65 years and over) showed similar time-lags between age-groups, though the time-lags tended to be slightly shorter for those aged 64 and under. In contrast, fitting the models to rounds 8 to 13 (30 December 2020 to 12 July 2021) of REACT-1 identified very different estimates between age groups and some extremely long time-lags. However, this was over a period of time when large proportions of the population were being vaccinated against Coronavirus Disease 2019 (COVID-19), with different rates of vaccination by age group (Fig 2). This could have led to continuously changing severity, severely biasing estimates obtained from the model. Due to the likely high degree of bias during rounds 8 to 13, below we present only the models obtained from rounds 1 to 7 and rounds 14 to 19.

**Fig 3:**
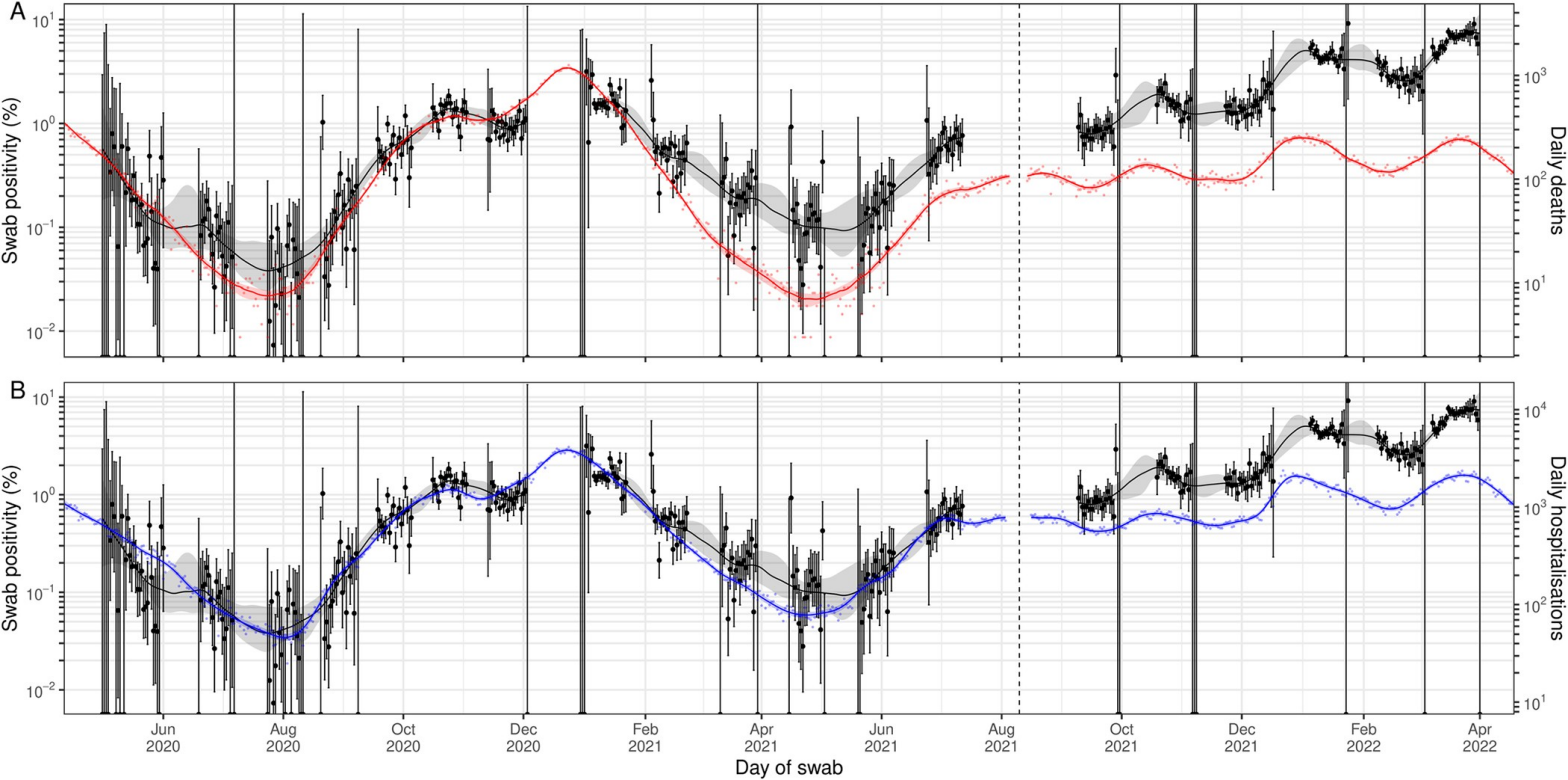
A comparison of daily deaths and hospitalisations to swab positivity as measured by REACT-1. Daily swab positivity for all 19 rounds of the REACT-1 study (black points with 95% credible intervals, left hand y-axis) with P-spline estimates for swab positivity (solid black line, shaded area is 95% credible interval). **(A)** Daily deaths in England (red points, right hand y-axis) and P-spline model estimates for expected daily deaths in England (solid red line, shaded area is 95% credible interval, right hand y-axis). The black vertical dashed line on 10 August 2021 splits the data into 2 periods: rounds 1–13 and rounds 14–19 of REACT-1. During rounds 1–13, daily deaths have been shifted by 26 days backwards in time along the x-axis. During rounds 14–19, daily deaths have been shifted by 18 days backwards in time along the x-axis. The 2 y-axes have been scaled using the population size and best-fit scaling parameter from the time-delay model fit to rounds 1–7. **(B)** Daily hospitalisations in England (blue points, right hand y-axis) and P-spline model estimates for expected daily hospitalisations in England (solid blue line, shaded area is 95% credible interval, right hand y-axis). The black vertical dashed line on 10 August 2021 splits the data into 2 periods: rounds 1–13 and rounds 14–19 of REACT-1. During rounds 1–13, daily hospitalisations have been shifted by 19 days backwards in time along the x-axis. During rounds 14–19, daily hospitalisations have been shifted by 7 days backwards in time along the x-axis. The 2 y-axes have been scaled using the population size and best-fit scaling parameter from the time-delay model fit to rounds 1–7 of REACT-1. Data supporting this figure can be found in S2 Data.

**Fig 4:**
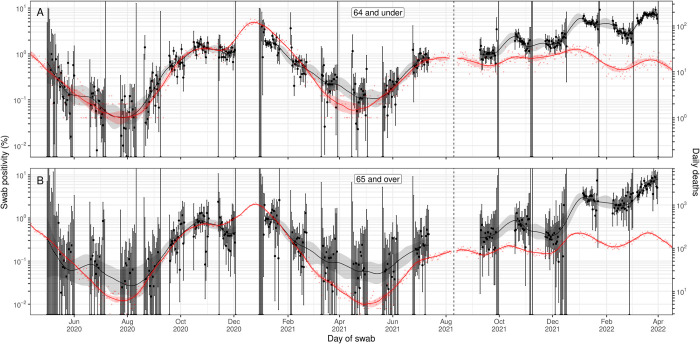
A comparison of daily deaths to swab positivity as measured by REACT-1, by age group. Daily swab positivity for all 19 rounds of the REACT-1 study (black points with 95% credible intervals, left hand y-axis) with P-spline estimates for swab positivity (solid black line, shaded area is 95% credible interval) for (**A**) those aged 64 years and under, and (**B**) those aged 65 years and over. **(A)** Daily deaths for those aged 64 years and under in England (red points, right hand y-axis) and corresponding P-spline model estimates for the expected number of deaths (solid red line, shaded area is 95% credible interval, right hand y-axis). The black vertical dashed line on 10 August 2021 splits the data into 2 periods: rounds 1–13 and rounds 14–19 of REACT-1. During rounds 1–13, daily deaths have been shifted by 24 days backwards in time along the x-axis. During rounds 14–19, daily deaths have been shifted by 16 days backwards in time along the x-axis. The 2 y-axes have been scaled using the population size and best-fit scaling parameter from the time-delay model fit to rounds 1–7 of REACT-1. **(B)** Daily deaths for those aged 65 years and over in England (red points, right hand y-axis) and corresponding P-spline model estimates for the expected number of deaths (solid red line, shaded area is 95% credible interval, right hand y-axis). The black vertical dashed line on 10 August 2021 splits the data into 2 periods: rounds 1–13 and rounds 14–19 of REACT-1. During rounds 1–13, daily deaths have been shifted by 24 days backwards in time along the x-axis. During rounds 14–19, daily deaths have been shifted by 19 days backwards in time along the x-axis. The 2 y-axes have been scaled using the population size and best-fit scaling parameter from the time-delay model fit to rounds 1–7 of REACT-1. Data supporting this figure can be found in S2 Data.

**Fig 5:**
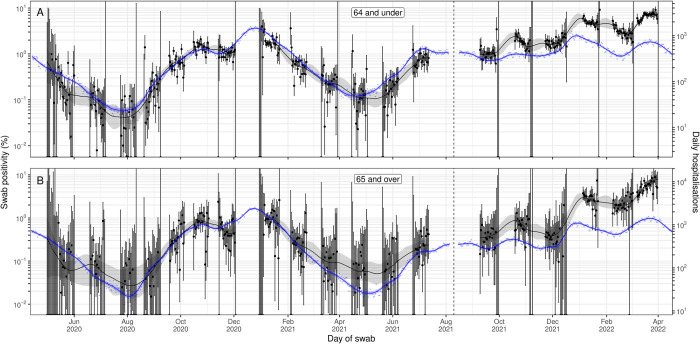
A comparison of daily hospitalisations to swab positivity as measured by REACT-1, by age group. Daily swab positivity for all 19 rounds of the REACT-1 study (black points with 95% credible intervals, left hand y-axis) with P-spline estimates for swab positivity (solid black line, shaded area is 95% credible interval) for (**A**) those aged 64 years and under, and (**B**) those aged 65 years and over. **(A)** Daily hospitalisations for those aged 64 years and under in England (blue points, right hand y-axis) and corresponding P-spline model estimates for the expected number of hospitalisations (solid blue line, shaded area is 95% credible interval, right hand y-axis). The black vertical dashed line on 10 August 2021 splits the data into 2 periods: rounds 1–13 and rounds 14–19 of REACT-1. During rounds 1–13, daily hospitalisations have been shifted by 17 days backwards in time along the x-axis. During rounds 14–19, daily hospitalisations have been shifted by 6 days backwards in time along the x-axis. The 2 y-axes have been scaled using the population size and best-fit scaling parameter from the time-delay model fit to rounds 1–7 of REACT-1. **(B)** Daily hospitalisations for those aged 65 years and over in England (blue points, right hand y-axis) and corresponding P-spline model estimates for the expected number of hospitalisations (solid blue line, shaded area is 95% credible interval, right hand y-axis). Daily hospitalisations have been shifted by 18 days backwards in time along the x-axis. The black vertical dashed line on 10 August 2021 splits the data into 2 periods: rounds 1–13 and rounds 14–19 of REACT-1. During rounds 1–13, daily hospitalisations have been shifted by 18 days backwards in time along the x-axis. During rounds 14–19, daily hospitalisations have been shifted by 9 days backwards in time along the x-axis. The 2 y-axes have been scaled using the population size and best-fit scaling parameter from the time-delay model fit to rounds 1–7 of REACT-1. Data supporting this figure can be found in S2 Data.

There was a high degree of correlation between smoothed estimates of swab positivity and the time-delayed signal of severe outcomes, though over the course of the study, the 2 signals substantially diverged (Figs 3–5, S2 Table). Assuming the estimated time-lag for the time-delay models fit to rounds 1 to 7 of REACT-1, we found a Pearson correlation coefficient for modelled swab positivity over rounds 1 to 7 against death and hospitalisations of 0.985 and 0.978, respectively (*p*-values < 0.001). Similarly high levels of correlation were found for rounds 8 to 13 assuming the same time-lag. Correlation over rounds 14 to 19 was lower at 0.732 and 0.757 (for death and hospitalisations, respectively) when assuming a time-lag from models fit to rounds 1 to 7 (though still significant, *p*-values < 0.001), but only slightly lower at 0.895 and 0.955 (for death and hospitalisations, respectively) when assuming the time-lags from models fit to rounds 14 to 19. Broadly similar patterns in correlations were found when looking at both age-groups. However, for those aged 64 and under during rounds 14 to 19, correlation was greatly reduced with a value of 0.169 (*p*-value = 0.01), even when estimating using the time-lag from the model fit to rounds 14 to 19.

The estimated severity of infection was found to decrease over the duration of the study. Using the time-delay model, we were able to estimate the IFR and IHR (see Methods for assumptions). Fitting the model to rounds 1 to 7 (1 May to 3 December 2020), we estimated the IHR to be 2.6% (2.5%, 2.7%), and the IFR to be 0.67% (0.65%, 0.70%). Fitting the model instead to rounds 14 to 19 (9 September 2021 to 31 March 2022), we estimated the IHR to be approximately 3.5-fold lower at 0.76% (0.75%, 0.77%), and the IFR to be approximately 7-fold lower at 0.097% (0.096%, 0.099%).

The severity of the virus, as measured per infected individual, was far lower in those aged 64 and under, relative to those aged 65 and over. From the models fitted to rounds 1 to 7 (1 May to 3 December 2020), we estimated the IHR to be 0.96% (0.93%, 1.00%) and the IFR to be 0.059% (0.057%, 0.061%) for those aged 64 and under. In comparison, the IHR and IFR for those aged 65 and over were approximately 16-fold and 91-fold higher, at 15% (14%, 17%) and 5.4% (4.9%, 5.9%), respectively, for the same time period. As before, estimates of the IHR and IFR were found to be lower when the model was fitted to rounds 14 to 19.

### Changes in severity during mass vaccination and the emergence of Alpha, Delta

**Fig 6:**
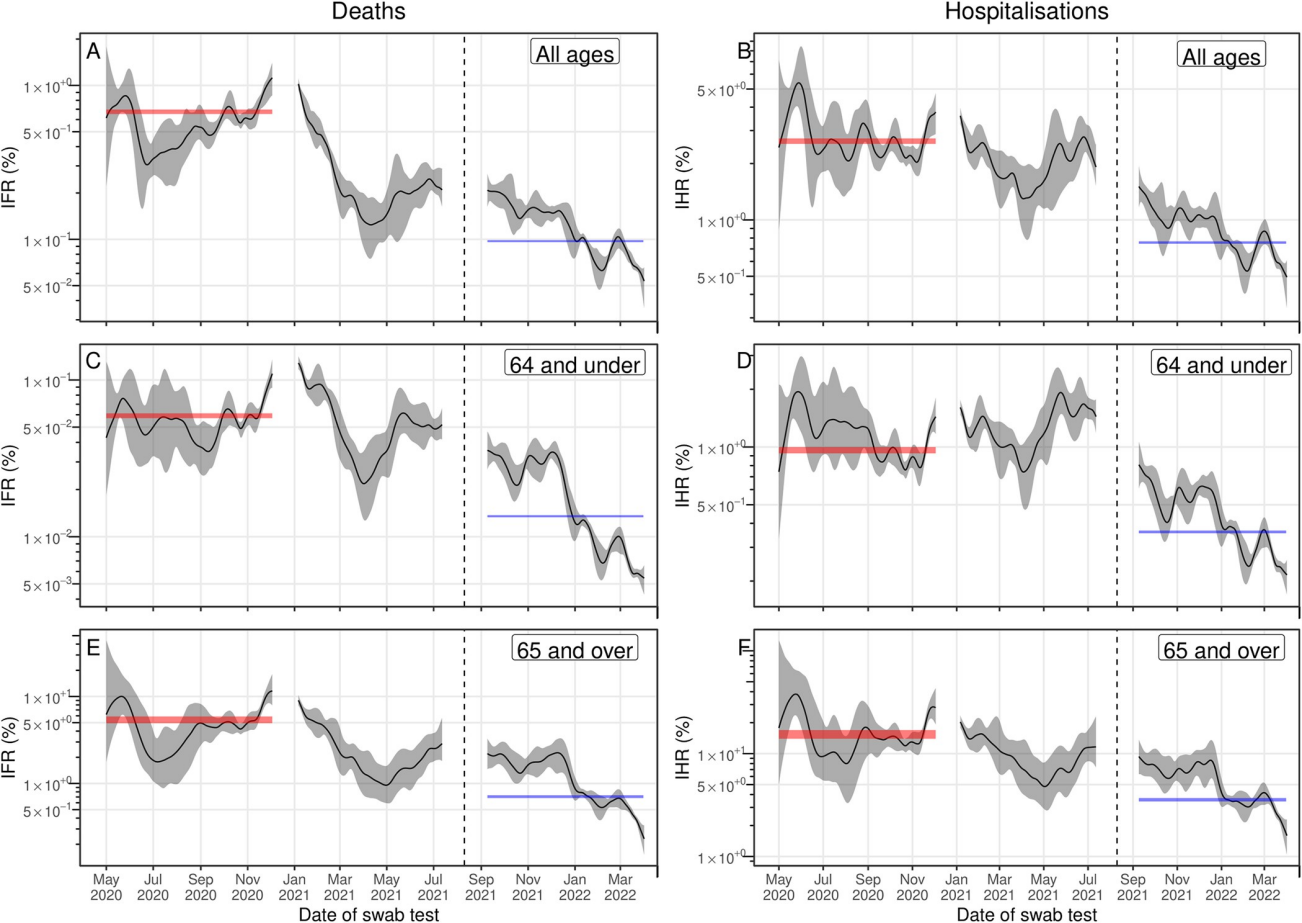
Estimates of the IFR and IHR over 19 rounds of REACT-1. IFR and IHR (solid black line, grey shaded region is 95% credible interval) estimated from the multiplicative difference between the REACT-1 P-splines for swab positivity and the time-delay adjusted death or hospitalisation P-splines, accounting for population size, mean duration of positivity, and test sensitivity. The 95% credible intervals of the best-fitting average IFR and IHR over rounds 1–7 (red shaded area) and rounds 14–19 (blue shaded area) estimated using time-delay models are shown for comparison (available in S1 Table). The black vertical dashed line on 10 August 2021 splits the data into 2 periods: rounds 1–13 and rounds 14–19 of REACT-1. (**A**) IFR across all age groups assuming a time-lag of 26 days during rounds 1–13 and 18 days during rounds 14–19. (**B**) IHR across all age groups assuming a time-lag of 19 days during rounds 1–13 and 7 days during rounds 14–19. (**C**) IFR in those aged 64 years and under assuming a time-lag of 24 days during rounds 1–13 and 16 days during rounds 14–19. (**D**) IHR in those aged 64 years and under assuming a time-lag of 17 days during rounds 1–13 and 6 days during rounds 14–19. (**E**) IFR in those aged 65 years and over assuming a time-lag of 24 days during rounds 1–13 and 19 days during rounds 14–19. (**F**) IHR in those aged 65 years and over assuming a time-lag of 18 days during rounds 1–13 and 9 days during rounds 14–19. Data supporting this figure can be found in S3 Data.

We detected an increase in the severity of infection into the winter of 2020. Using the best-fit time-lag obtained from fitting the time-delay model to rounds 1 to 7 (1 May to 3 December 2020) of REACT-1, we estimated the daily IFR and IHR from the modelled estimates of swab positivity and the time-lag adjusted signals of hospitalisations and deaths over rounds 1 to 13 (1 May 2020 to 12 July 2021) (Fig 6). An increase in the daily IFR was observed in late November 2020, with the increase lasting until late January 2021. We compared the mean IFRs and IHRs over approximately 4-week periods to a baseline period from 1 May 2020 to 11 November 2020 (S3 Table), a period before vaccinations when wild-type variants dominated. The mean IFR in late November 2020 (8 November to 5 December) was 1.68 (1.39, 1.93) times greater than baseline, and in January 2021 (3 January to 30 January) was 1.31 (1.11, 1.56) times greater than baseline; during these 2 periods, the Alpha variant was responsible for 15% and 86% of infections, respectively. The increase in the IFR was observed in both age-groups, but no increase was observed in the IHR.

The severity of the virus, as measured by the IFR and IHR, decreased from late January 2021 until April 2021, after which it increased until 12 July 2021 (the end of round 13). Both the daily IFR and daily IHR decreased until April. The mean IFR in April 2021 (28 March to 24 April) reduced to 0.25 (0.17, 0.34) of baseline, and the mean IHR to 0.51 (0.35, 0.68) of baseline. During this period, the Alpha variant represented 96% of infections, 47% of the population had received at least 1 dose, and 11% 2 doses of a vaccine. By June/July 2021 (20 June to 17 July), the mean IFR increased to 0.43 (0.37, 0.53) of baseline, and the mean IHR increased to 0.84 (0.72, 1.03) of baseline. At this point, while the proportion vaccinated had increased to 66% with at least 1 dose and 50% with 2 doses, the Delta variant was now the dominant variant representing 99% of infections over this period.

There was a more substantial and quicker decrease in severity for those aged 65 and over, compared to those aged 64 and under. In April 2021 (28 March to 24 April), for those aged 65 and over, the mean IFR and IHR were 0.28 (0.18, 0.49) and 0.42 (0.27, 0.74) of baseline, respectively. For those aged 64 and under, the mean IFR and IHR were comparatively higher (though with overlapping credible intervals) at 0.48 (0.33, 0.71) and 0.72 (0.52, 0.97) of their baseline, respectively. By June/July 2021 (20 June to 17 July), the mean IFR had increased to 0.98 (0.80, 1.15) of baseline for those aged 64 and under, but only to 0.63 (0.44, 1.06) for those aged 65 and over. The mean IHR in June/July 2021 had only increased to 0.76 (0.53, 1.24) for those aged 65 and over, but it was now significantly higher than baseline in those aged 64 and under, at 1.32 (1.14, 1.55) times baseline. There were large differences in the proportion vaccinated by this period; 98% of those aged 65 and over had received 2 doses of vaccine, whereas only 39% of those aged 64 and under had received 2 doses of vaccine.

### Changes in severity during booster vaccination and the emergence of Omicron

From September 2021 to April 2022, the severity of infection decreased. Using the best-fit time-lag obtained from fitting the time-delay model to rounds 14 to 19, we estimated the daily IFR and IHR from the modelled estimates of swab positivity and the time-lag adjusted signals of hospitalisations and deaths for rounds 14 to 19 of REACT-1 (Fig 6). The daily IFR and IHR decreased steadily from September 2021 onwards with a sharp and rapid reduction in late December 2021. Over this period, the proportion of the population that had received a third dose of vaccine (“booster” dose) steadily increased, saturating at about 54% by mid-January 2021 (S4 Table). Observed trends in the daily IFR and IHR were broadly similar across both age groups, despite approximately double the proportion of the population aged 65 and over having received a booster dose, compared to the population aged 64 and under, by the end of March 2022. The rapid reduction of the daily scaling parameters in late December 2021 coincided with the rapid increase in the proportion of infections caused by the Omicron variant. We compared the mean IFRs and IHRs over approximately 4-week periods to a baseline period from 4 September 2021 to 16 October 2021 (S4 Table), a period before Omicron’s emergence and with low proportions of the population having received booster doses. By March 2022 (6 March to 2 April), when Omicron had reached near total coverage (responsible for 99% of infections), the mean IFR was 0.069% (0.066%, 0.072%) at 0.36 (0.31, 0.42) of baseline, and the mean IHR was 0.62% (0.58%, 0.65%) at 0.50 (0.44, 0.59) of baseline.

### Changes in the case ascertainment rate of England’s mass testing programme

**Fig 7:**
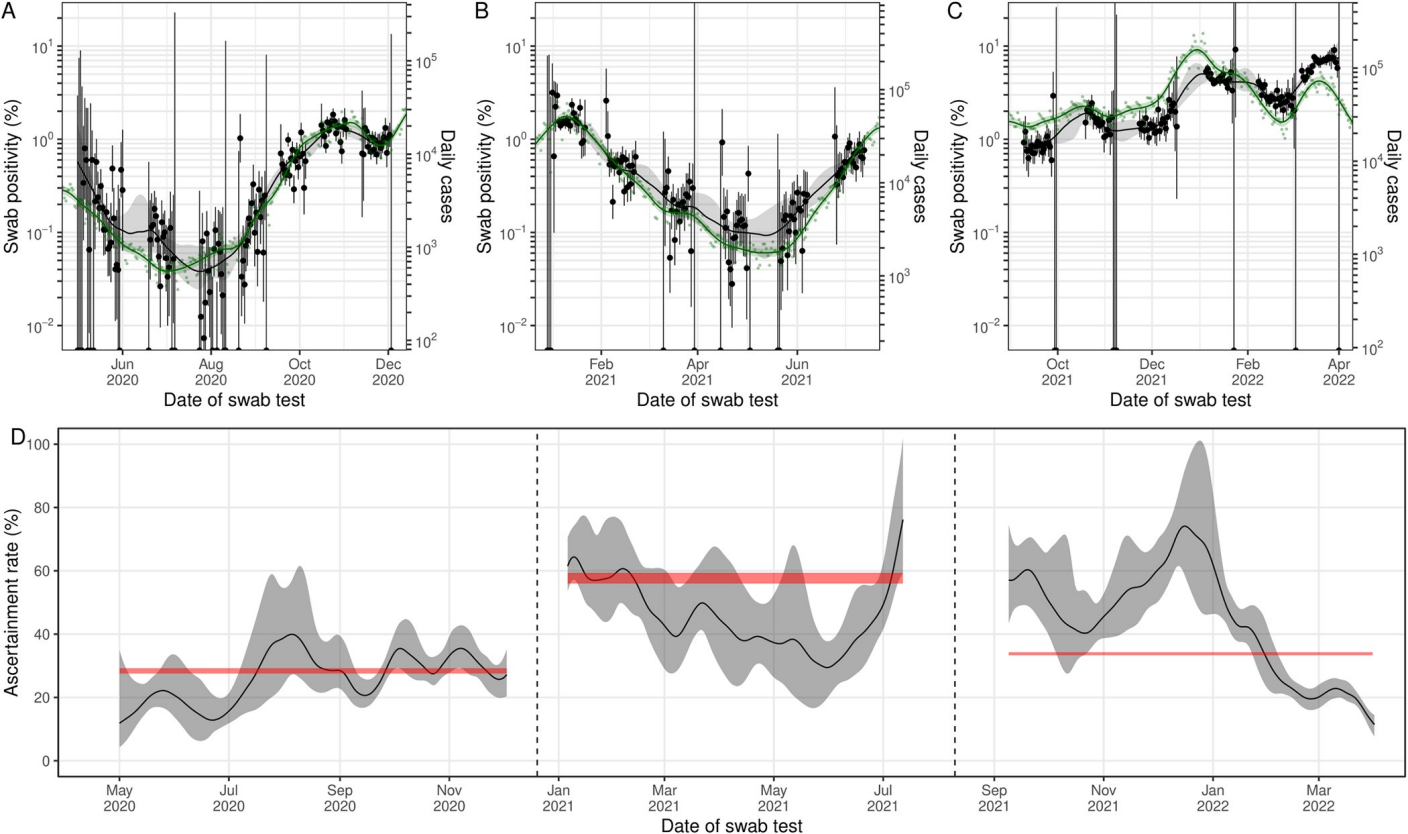
A comparison of daily cases to swab positivity as measured by REACT-1. (**A**, **B**, **C**) Daily swab positivity for all 19 rounds of the REACT-1 study (black points with 95% credible intervals, left hand y-axis) with P-spline estimates for swab positivity (solid black line, shaded area is 95% credible interval). Daily cases in England (green points, right hand y-axis) and P-spline model for expected daily cases in England (solid green line, shaded area is 95% credible interval, right hand y-axis). The 2 y-axes have been scaled using the population size and best-fit scaling parameter from the time-delay model fit to the rounds shown in each subfigure (available in S5 Table). (**A**) During round 1–7, daily cases have been shifted by 3 days backwards in time along the x-axis. (**B**) During round 8–13, daily cases have been shifted by 7 days forwards (−7 days backwards) in time along the x-axis. (**C**) During round 14–19, daily cases have been shifted by 1 day backwards in time along the x-axis. (**D**) Estimates of the case ascertainment rate over 19 rounds of REACT-1. Case ascertainment (solid black line, grey shaded region is 95% credible interval) estimated from the multiplicative difference between the REACT-1 P-spline for swab positivity and the time-delay adjusted P-spline for daily cases, accounting for population size, mean duration of positivity, and test sensitivity. The 95% credible intervals of the best-fitting average case ascertainment rates (red shaded area) over rounds 1–7, rounds 8–13, and rounds 14–19 estimated using separate time-delay models are shown for comparison (available in S5 Table). The black vertical dashed lines split the data into 3 periods: round 1–7, rounds 8–13, and rounds 14–19 of REACT-1. During rounds 1–7, the case ascertainment was estimated assuming a time-lag of 3 days. During rounds 8–13, the case ascertainment was estimated assuming a time-lag of −7 days. During rounds 14–19, the case ascertainment was estimated assuming a time-lag of 1 days. Data supporting this figure can be found in S4 Data.

The time-lag between swab positivity and daily case numbers (identified through England’s mass testing) varied over the duration of the study. Fitting the time-delay model (see Methods) for daily case numbers to rounds 1 to 7 (1 May to 3 December 2020) of REACT-1 we estimated a discrete time-lag of 3 (3, 4) days (Fig 7, S5 Table). Over rounds 8 to 13 (30 December 2020 to 12 July 2021), we estimated a discrete time-lag of −7 (−8, −7) days (the time-series of daily cases led the time-series of swab positivity), and over rounds 14 to 19 (9 September 2021 to 31 March 2022), we estimated a discrete time-lag of 1 (1, 1) days. There was a high degree of correlation between the smoothed estimates of swab positivity and the time-adjusted signals of daily case numbers, but this correlation was lowest for rounds 14 to 19, which saw some divergences in the 2 signals (Fig 7, S6 Table).

The estimated case ascertainment rate (the proportion of infections identified with a positive test by England’s mass testing programmes [see Methods]) varied over the duration of the study. Similarly to before (when estimating IFR and IHR), we were able to use the time-delay model to estimate the case ascertainment rate (see Methods for assumptions). Fitting the model to all 19 rounds, we estimated the case ascertainment rate to be 36.1% (35.7%, 36.5%). However, fitting the model instead to subsets of rounds, we identified changes in the case ascertainment rate. Fitting the model to rounds 1 to 7, we estimated the case ascertainment rate to be 28.3% (27.5%, 29.2%). Fitting the model to rounds 8 to 13, we estimated the case ascertainment rate to be higher at 57.6% (56.0%, 59.4%). Finally, fitting the model to rounds 14 to 19, we estimated the case ascertainment rate to have decreased to 33.8% (33.3%, 34.3%).

There were some clear temporal trends in the daily case ascertainment rate at the timing of key changes. Using the best-fit time-lags obtained from fitting the time-delay model to each period of the REACT-1 study (rounds 1 to 7, 8 to 13, and 14 to 19), we estimated the daily case ascertainment rates from the modelled estimates of swab positivity and the time-lag adjusted daily case numbers (Fig 7). The case ascertainment rate increased in July 2020 from approximately 20% (for the period of May to June 2020) to approximately 30% (for the period of August to December 2020), in line with the rollout of mass community testing in England on 2020 July 2 (previously testing was clinically focused) [6]. There was a sharp increase in the case ascertainment rate from May 2021 to July 2021, a period in which Delta had just replaced Alpha as the dominant variant. In contrast, there was a sharp decrease in the case ascertainment rate from December 2021 to March 2022, the period in which Omicron replaced Delta as the dominant variant and resulted in 2 large waves of infection prevalence in England.

## Discussion

We show a clear temporal relationship between prevalence of SARS-CoV-2 infection in the community, as measured by swab positivity, and severe outcomes, suggesting that in the future, large community testing studies such as REACT-1 could be used not just to estimate prevalence but also for short-term forecasting of severe outcomes. Previous analysis has suggested the time-lag between symptom onset and hospitalisations to be approximately 8 days [19,32,33] and between symptom onset and death to be approximately 16 days [18,19]. In REACT-1, our estimates, during rounds 1 to 7 (May 2020 to December 2020), of the time-lag between swab positivity and these severe outcomes were higher, possibly because REACT-1 better captured asymptomatic and presymptomatic infections due to its random, community-based sampling procedure. Additionally, during rounds 1 to 7 and rounds 14 to 19 of REACT-1, the time series of swab positivity led the time series of daily cases (as identified through England’s mass testing programme), suggesting that swab positivity was a better approximation to infection incidence than daily cases over these periods. However, during rounds 8 to 13 of REACT-1, swab positivity lagged daily cases by about a week.

During rounds 14 to 19 (September 2021 to March 2022), the time-lag between REACT-1 swab positivity and severe outcomes was shorter and more in line with previous studies based on symptom onset. This could suggest a change in the inherent biology of the virus, due to the emergence of the Omicron variant and/or the substantial buildup of immunity (due to vaccination and natural infection). In addition, the extremely high levels of prevalence observed in England over this period [17] mean that large numbers of individuals may have been hospitalised (or died) “with” SARS-CoV-2 infection rather than “from.” Thus, the time series of severe outcomes may be a mixture of 2 components, the true signal (where infection resulted in a severe outcome) and a pseudo-signal reflecting the high prevalence of infection in the population. This would have the effect of reducing the time-lag between infection and hospitalisation/death and may also explain the lower levels of correlation observed during this period.

In January 2021, the government’s mass vaccination campaign was substantially accelerated. Vaccines have been shown to be highly effective in preventing deaths related to COVID-19 [8], and so it is unsurprising that a few weeks after this, the rate of deaths began to diverge from prevalence. This “decoupling” was earlier and more pronounced in those aged 65 and over, most likely reflecting the vaccination campaign prioritising the oldest individuals first. Though some degree of “decoupling” was seen in the hospitalisation data, it was less evident and occurred later. A possible explanation is that the vaccine has led to an overall reduction in the “severity pyramid” of the virus, with those who might have died if unvaccinated, instead only hospitalised, delaying the reduction in hospitalisations. Another potential explanation is that the early vaccination of healthcare workers could have led to a reduction in nosocomial transmission leading to fewer infections in vulnerable patients and fewer deaths.

By measuring the difference between smoothed estimates of swab positivity, and the time-lag adjusted signals for deaths, hospitalisations, and cases, we were able to estimate the IFR and IHR of the SARS-Cov-2 virus and the case ascertainment rate of England’s mass testing over time. Though these estimates relied on some assumptions (see Methods), they showed strong agreement with other available estimates. We estimated an IHR of 2.6% during rounds 1 to 7 of REACT-1, compared to an estimated IHR at the start of England’s first wave of 2.55% [23]. Our estimate of IFR over rounds 1 to 7 at 0.67% was slightly lower than the estimated IFR at the start of England’s first wave (1.00%), but consistent with that at the end of the first wave of 0.79% (0.63%, 0.99%) [23]. Over the entire 19 rounds of REACT-1, we estimated the case ascertainment rate in England to be 36.1%, which is in line with other work that has estimated case ascertainment in England to be between 20% and 40% over this period [34].

There are large amounts of noise and correlation in the continuous estimates of the IFR and IHR, making it difficult to assess overall trends. However, there does appear to have been a reduction in the IFR over the summer of 2020 as reported in previous analysis [23]. There also appears to have been an upwards trend in the IFR and IHR into January 2021, as previously reported [35], potentially driven by increased severity of the Alpha variant [5], seasonality [36], or increased pressure on health services. However, the REACT-1 study was not in the field for most of December 2020 so is poorly placed to identify this as an estimate for the December peak in prevalence could not be made. Overall hospitalisations appear to have more closely followed prevalence of infections than did deaths. This suggests a nonlinearity between hospitalisations and deaths that could be a product of patterns in transmission, changing standards of care, changing criteria for admissions, or propensity to seek care.

The rapid increase in both the IFR and IHR from April 2021 likely reflects the emergence of the Delta variant and its rapid spread during a period with few new vaccinations among the most at-risk groups [11]. Vaccine effectiveness against Delta was lower than for previously circulating lineages after a single dose but broadly similar in those who had been double-vaccinated [13]. This could explain the differences between age groups, with the IFR and IHR increasing to a greater extent in those aged 64 years and under, as by the end of April 2021, there was near total coverage of those aged over 65 with 2 doses of the vaccine. By July 2021, Delta accounted for almost all infections [12], and vaccine coverage in those aged over 65 years was approximately constant. At this point, the IFR for those aged over 65 years was higher than the lowest estimate in April 2021, suggesting that the Delta variant resulted in increased disease severity over Alpha and other previously circulating lineages. It has previously been estimated that there is an approximately 2-fold greater hazard of hospitalisation when infected with the Delta variant relative to Alpha [37,38], which could explain the large increase in the IHR even with high vaccine coverage. Following Delta’s emergence, the case ascertainment rate was found to sharply increase from May 2021 to July 2021; this increase has been reported elsewhere [34] and could be due to higher rates of symptoms in Delta infected individuals (more likely to test symptomatic individuals) or due to confounding behavioural factors such as more lateral flow tests being used by individuals over this period.

A combination of booster vaccine doses and the emergence of the Omicron variant likely contributed to the reduction in both the IFR and IHR from September 2021 to March 2022. While booster doses are highly effective at reducing the odds of death or hospitalisation [39,40], their contribution to reducing the IHR and IFR is unknown (though there appeared to be small reductions in the IFR and IHR over the period booster doses were administered). Without booster doses, there may have been an increase in the IFR and IHR due to waning of vaccine effectiveness [25,39]. Omicron infections lead to less severe disease than Delta infections [41], which may explain the rapid reduction of the IFR and IHR in December 2021 (when Omicron rapidly replaced Delta as the dominant variant). Similarly, there was a rapid reduction in the case ascertainment rate from December 2021 to March 2022. This could be a result of the saturation of testing capacity due to the high infection levels caused by the Omicron variant over this period; for example, there were shortages of lateral flow tests in England in December 2021 and January 2022 due to increased demand.

Our study has limitations. We required an extensive run of REACT-1 data to obtain an accurate estimate of the time-lag parameter, which restricts our ability to detect changes in the time-lag over shorter periods. It is also possible that the vaccine programme and emergence of new variants may have affected the time-lag between infection and hospitalisations and deaths in a way not accounted for in the analysis. Additionally, in estimating the time-lag parameter, we have also had to assume that there have been no substantial changes in the IFR and IHR over the period the model is fitted to; fluctuations in the IFR and IHR will have little effect, but if they change steadily over time (in a single direction [up or down]), then the estimates of the time-lag might be skewed. However, when we assume that the time-lag parameter is a known constant, we see that the IFR and IHR have both changed substantially over time; this limitation was demonstrated with the model fit to rounds 8 to 13, which saw implausibly long time-lags with massive variation in the time-lags between age groups. Rounds 8 to 13 likely saw the greatest changes in the relationship between infection and severe outcomes due to England’s mass vaccination programme.

REACT-1 tests for swab positivity and not infection prevalence. When estimating the IFR, IHR, and case ascertainment rate, we have had to convert our modelled estimates of swab positivity into estimates of infection prevalence. Our conversion relies on a simple assumption that the two are related by a constant multiplicative factor, composed of the mean duration of positivity (14.0 days) and the sensitivity of the swab tests (79%) [42]. However, it is likely that these 2 values have changed over time with the introduction of new variants and high rates of vaccination. Over periods in which these 2 values are constant, the temporal dynamics we present for the IFR, IHR, and case ascertainment will be correct, but the magnitude may be off by a constant factor. Additionally, as REACT-1 tested for swab positivity, long-term shedders [43] may have inflated the estimates of infection rate, especially for periods of low prevalence following periods of high prevalence.

While REACT-1 provides an accurate picture of prevalence in the community, severe outcomes may be more closely linked to prevalence in at-risk individuals. For example, trends in prevalence in care homes during the pandemic showed some marked differences from community prevalence [23], which may have introduced biases. Though our time-delay model assumes a straightforward time-lag between prevalence and severe outcomes (a convolution with a delta function), a transformation involving a convolution with a more dispersed shape is perhaps more realistic. This may lead to trends in prevalence over short time-scales being smoothed out in the resulting death and hospitalisation time series.

## Conclusions

Over the duration of the pandemic in England, there has been a decline in the severity of infections caused by the SARS-CoV-2 virus, reflecting both high rates of vaccination in the community and the emergence of the Omicron variant. However, there remain many populations worldwide that have lower vaccination rates than in England, and where severity of illness may not have reduced to as great an extent. In addition, it is possible that the emergence of future variants may lead to more severe disease, as was observed for the Alpha and Delta variants. In preparing for future waves of SARS-CoV-2 infection, it remains paramount that any increases in severity are detected rapidly. Community-based prevalence studies such as REACT-1 can provide unbiased estimates of infection levels over time, allowing any changes in the IFR or IHR to be identified quickly. With advanced warning, appropriate interventions can then be implemented when they are most effective, for example, provision of additional vaccine doses and enhanced healthcare measures.

## Methods

### REACT-1 data

The REACT-1 methods are published [17,29]. At each round, a random subset of the population aged 5 years and over was contacted by letter using the list of general practitioner patients in England held by the NHS. Those who agreed to participate were then sent a self-administered swab test (for 5- to 12-year-olds, a parent/guardian administered the test). The participants completed a questionnaire providing sociodemographic information such as age, gender, ethnicity, and occupation. Swab tests were collected by a courier and sent via cold chain (latterly by post) to a commercial laboratory for a reverse transcription polymerase chain reaction (RT-PCR) test for SARS-CoV-2. A test was swab positive if either both N- and E-gene were detected, or N-gene detected with a Ct value less than 37. The REACT-1 study received research ethics approval from the South Central-Berkshire BResearch Ethics Committee (IRAS ID: 283787). Consent was obtained from all participants or their parent/guardian for minors. During initial registration for the study, participants were asked “Are you willing to take part in this study?/Are you willing for your child to take part in this study?” with possible answers being “1. Yes, I want to take part in this study” or “2. No, I do not want to take part.”. Those who answered “2. No, I do not want to take part.” were not sent testing kits and did not participate further in the study.

### Public data

Data for deaths, hospitalisations, and cases were obtained from “the official UK government website for data and insights on coronavirus” [6]. Daily hospitalisation figures include all people in England who are admitted to hospital within 14 days of testing positive for SARS-CoV-2 and those who test positive after admission. Daily death figures include the number of people in England that died within 28 days of their first positive test for COVID-19 being reported (by date of death not date of reporting). Daily case numbers include the number of people in England who tested positive for SARS-CoV-2 by the date in which the sample was taken from the individual being tested. Hospitalisation and death data were available by age group; from this, we created 3 time series for each data set: the total number of events (death or hospitalisation), the number in those aged 64 years and under, and the number in those aged 65 years and over. Death, hospitalisation, and case data were downloaded on 20 May 2022, and only data up to 15 May 2022 were included in the analysis.

The daily cumulative number of individuals who had been vaccinated with a single dose and with 2 doses (any vaccine approved in the UK), by age group, was again downloaded from “the official UK government website for data and insights on coronavirus” [6]. Data by age group were collated into those aged 64 years and under, those aged 65 years and over, and at all ages. The cumulative number of vaccinations given (first and second doses) was then converted into the cumulative proportion of the population vaccinated using population estimates of England by age group [44]. Note that current population estimates are unavailable and are most likely greater than the values used; estimates of the proportion of the population vaccinated are thus not exact.

The weekly numbers of each SARS-CoV-2 lineage detected in routine surveillance data by lower tier local authority (LTLA) was downloaded from the “Lineage in Space and Time website” [45]. Data by LTLA were aggregated in order to give the daily number of each lineage detected in England as a whole. The weekly proportion of lineages that were the Alpha variant (or an Alpha sublineage), the Delta variant (or a Delta sublineage), and the Omicron variant (or an Omicron sublineage) was then straightforwardly calculated.

### P-spline model + mixed-effects P-spline

Smoothed models were fit to each time series in order to get an estimate of the expected number of outcomes (deaths, hospitalisations, and cases) or the expected prevalence (REACT-1 swab positivity). Bayesian P-spline models [46,47] were fit to the hospitalisation and death data for each age group (64 years or under, 65 years and over, and all ages), daily case data (all ages), and to the overall swab positivity for REACT-1. To maintain statistical power, a single mixed-effects Bayesian P-spline model was fit to the swab positivity in REACT-1 for the 2 age groups. The models consist of a system of basis splines defined over the window of the study period with approximately 1 basis spline every 5 days. The P-spline model is then a linear combination of these basis splines:

$$g(\pi_{j})=\Sigma_{i}b_{i}B_{ij},$$

where *π*_*j*_ is the outcome variable of interest on the *j*^*th*^ day, *g*() is the link function (logit for Binomial swab positivity data, log for count data), *b*_*i*_ is the coefficient for the *i*^*th*^ basis spline, and *B*_*ij*_ is the value of the *i*^*th*^ basis spline on the *j*^*th*^ day. Overfitting of the model was prevented through the inclusion of a second-order random walk prior distribution on the basis spline coefficients, *b*_*i*_:

$$b_{i}=2b_{i-1}-b_{i-2}+u_{i},$$

where

$$u_{i}∼N(0,\rho^{2}).$$

This prior distribution penalises any changes in the first derivative of the response function, reflecting the expected trend of an epidemic over a small time period (constant growth rate). The degree to which changes in the first derivative are penalised is controlled by *ρ*, which is a further parameter of the model and takes a loose but proper inverse gamma prior distribution *ρ*~*IG*(0.001,0.001). The first 2 basis spline coefficients are given an uninformative prior distribution, *b*_1_, *b*_2_~*Constant*.

In the mixed-effects version of the model in which the model fits to the time series for 2 age-groups simultaneously, the second-order random-walk prior distribution is modified slightly:

$$b_{i,k}=2b_{i-1,k}-b_{i-2,k}+u_{i,k},$$

where

$$u_{i,k}∼N(0,\rho^{2})$$

and

$$u_{i,k}∼N(\underline{u_{i,k}},\zeta^{2}),$$

where *u*_*i*,*k*_ is the mean value of *u*_*i*,*k*_ averaged over *k* age-groups. For the most part, this is the same as before, but now parameters are defined for *k* age-groups. The difference lies in that as well as penalising changes in the first derivative of the response function; differences between age groups in the changes of the first derivative are also penalised. This has the effect of syncing the model across age-groups while also allowing divergences to occur when there is sufficient evidence. The degree of penalisation is controlled by a further parameter *ζ*, which we give a loose but proper inverse-gamma prior distribution as before, *ζ*~*IG*(0.001, 0.001). All models are fit to data using a No-U-Turn Sampler (NUTS) [48] implemented in STAN [49]. For the REACT-1 data, we fit the daily weighted number of positive and negative tests assuming a Binomial likelihood. When fitting to the daily number of deaths, hospitalisations, and cases, we assume a Negative-Binomial likelihood with an overdispersion parameter that is treated as an additional parameter of the model with an uninformative constant prior distribution. The model fitting returns a full posterior distribution of all parameters from which the mean response function and credible intervals can be calculated. Estimates of swab positivity for the period between REACT-1 rounds 7 and 8 are not included in any analysis, as between the rounds, there was a large peak in infections that we cannot estimate using the REACT-1 data. Similarly, we did not include estimates of swab positivity for the period between rounds 13 and 14 in any analysis; this is because there was a substantial break in the study (approximately 2 months) for which dynamics cannot be inferred accurately. Estimates of swab positivity between other rounds are included as there was only a small break between most rounds (approximately 2 weeks).

### Time-delay model

In order to investigate the relationship between the REACT-1 data and hospitalisations, deaths, and cases, we define a simple model consisting of 2 parameters. The first parameter, the time-lag *τ*, sets the discrete number of days between the REACT-1 data and the time series of interest. The second parameter, the scaling factor *ϵ*, sets the multiplicative difference between REACT-1 data and the time series of interest correcting for population size. The estimated proportion swab positive on day *i* is then related to the time series of interest on day *i*+*τ* by:

$$(Proportion\;swab\;positive{)}_{i}\times Population\times \epsilon \times 0.01=(Time\;Series{)}_{i+\tau }.$$

Written this way, the scaling parameter, *ϵ*, represents the percentage of those in the population that are swab positive on day *i* that will be admitted to hospital, have died, or have tested positive for SARS-CoV-2 through mass testing on day *i*+*τ*. The modelled proportion swab positive on day *i*,

$$(Proportion\;swab\;positive{)}_{i}=(Time\;Series{)}_{i+\tau }\times 100/Population\times \epsilon ,$$

can then be fitted to the REACT-1 daily weighted number of positive and negative tests, assuming a weighted Binomial likelihood. If this was done using the raw data for hospitalisations, deaths, or cases, then there would be errors for any days in which zero counts occurred. There would also be the possibility for overfitting due to well-aligned noise between the raw time series and the REACT-1 data. In order to avoid these pitfalls, instead of using the raw data as the time series, the P-spline model estimates are used instead. To take into account the uncertainty in the P-spline model, a random subset of 1,000 parameter combinations is selected from the posterior distribution. For each set of parameter combinations, a time-series can be calculated. The average log-likelihood, fitting to the REACT-1 data, over all 1,000 random draws of the P-spline’s posterior distribution is then used to fit the time-lag and scaling parameter using an MCMC. The time-delay models were fit to all rounds of REACT-1 and to subsets of rounds: rounds 1 to 7, rounds 8 to 13, and rounds 14 to 19. Further analyses for deaths and hospitalisations were only performed using the time-lags from models fit to rounds 1 to 7 and rounds 14 to 19. Further analyses for daily cases were performed using the time-lags from models fit to rounds 1 to 7, rounds 8 to 13, and rounds 14 to 19. This was because drastic changes in severity over rounds 8 to 13 (due to vaccination) should not have had a large impact on case ascertainment over this period but would have had a large impact on the IFR and IHR.

### Correlation between time series

Under the assumption of a particular time-lag, the Pearson correlation was measured between the mean daily swab positivity (as estimated using the P-spline model) and the time-delayed mean daily outcomes (deaths, hospitalisations, or cases) (as estimated using the P-spline model). Correlation between swab positivity and daily outcomes was estimated for the periods of rounds 1 to 7, rounds 8 to 13, and rounds 14 to 19. For deaths and hospitalisations, the time-lags used were those estimated from the time-delay model fit to rounds 1 to 7 and fit to rounds 14 to 19. For daily cases, the time-lags used were those estimated from the time-delay model fit to rounds 1 to 7, rounds 8 to 13, and rounds 14 to 19.

### Variation in scaling parameter over time

With the assumption of a particular time-lag, and that it does not change over time, variation in the scaling parameter over time between REACT-1 data and hospitalisation, death, or case data can straightforwardly be estimated. For each day, the posterior distribution for the estimate of swab positivity that day, inferred from the P-spline model fit to REACT-1, can be extracted. Similarly, the posterior distribution for the estimate of hospitalisations, deaths, and cases on a fixed number of days later (determined by time-lag) can also be extracted. The daily scaling parameter, and its uncertainty, between the 2 P-spline estimates can then be calculated for the whole study period.

We calculated the average scaling parameter over a set period of time for deaths and hospitalisations. We split the time period of rounds 1 to 13 of REACT-1 into approximately 4-week periods and 1 initial baseline period running from 1 May 2020 to 7 November 2020. This period was chosen as the baseline as it was before any vaccination had occurred and before the Alpha variant had increased to a substantial proportion. Similarly, we split the time period of rounds 14 to 19 of REACT-1 into approximately 4-week periods and 1 initial baseline period running from 4 September 2021 to 16 October 2021. This period was chosen as the baseline as it occurred before Omicron emerged and before a substantial proportion of the population had received a third vaccine dose.

For each period, we extracted the posterior distribution for the estimates of swab positivity over the period, inferred from the P-spline model fit to REACT-1, and calculated the posterior distribution of the mean swab positivity over the period. Similarly, we extracted the posterior distribution for the estimate of hospitalisations and deaths for the equivalent period a fixed number of days later (determined by the time-lag) and calculated the posterior distribution of the mean. We then estimated the mean scaling parameter (the difference between mean swab positivity and mean hospitalisations or deaths) over the period, together with its uncertainty. Additionally, we estimated the multiplicative difference between the mean scaling parameter for a specific period and the baseline period and its uncertainty.

### Converting the scaling parameters to the IFR, IHR, and case ascertainment rate

The scaling parameters above correspond to the percentage of those who are swab positive in a population on a particular day who will be hospitalised/have died/tested positive (as a result of mass testing) on a day in the future set by the time-lag parameter. By converting swab positivity to the incidence of infection, we can use the scaling parameters to estimate the IFR, IHR, and case ascertainment rate. The time-series of incidence and swab positivity are similar—though with a time-delay due to the finite duration, individuals remain swab positive after infection [42]. Under the simplifying assumption that swab positivity can be converted to incidence by a multiplicative constant, the IFR, IHR, and case ascertainment rate can be estimated by multiplying the scaling parameter by the same constant; we use the mean duration that an individual remains swab positive, estimated at 14.0 days, and sensitivity to detect a positive swab, estimated at 0.79 [42]. These 2 numbers allow all scaling parameter estimates to be converted to IFR (for deaths) and IHR (for hospitalisations) by multiplying the scaling parameter by 14.0×0.79 = 11.06.

## Supporting information

S1 Table Estimated time-lags and IFR and IHR for all time-delay models fit to REACT-1. (XLSX)

S2 Table Pearson correlation between modelled estimates of swab positivity and modelled estimates of deaths and hospitalisations. (XLSX)

S3 Table The average proportion of population vaccinated, average proportion of infections caused by each variant, and mean IFR and IHR over fixed periods of time over the duration of rounds 1–13 of REACT-1. Mean IFR and IHR for each period are compared to a baseline period running from 1 May 2020–7 November 2020. The time-lags between positivity and deaths and hospitalisations used in calculating mean IFR and IHR are the best-fit time-lags from the time-delay models fit to rounds 1–7 of REACT-1.
(XLSX)

S4 Table The average proportion of the population vaccinated, average proportion of infections caused by each variant, and mean IFR and IHR over fixed periods of time over the duration of rounds 14–19 of REACT-1. Mean IFR and IHR for each period are compared to a baseline period running from 4 September 2021–16 October 2021. The time-lags between positivity and deaths and hospitalisations used in calculating mean IFR and IHR are the best-fit time-lags from the time-delay models fit to rounds 14–19 of REACT-1.
(XLSX)

S5 Table Estimated time-lags and case ascertainment for time-delay models fit to REACT-1 using the time series of daily cases. (XLSX)

S6 Table Pearson correlation between modelled estimates of swab positivity and modelled estimates of daily cases. (XLSX)

S1 Data Supporting data for Fig 2. (XLSX)

S2 Data Supporting data for Figs 3, 4 and 5. (XLSX)

S3 Data Supporting data for Fig 6. (XLSX)

S4 Data Supporting data for Fig 7. (XLSX)

## Abbreviations

COVID-19: Coronavirus Disease 2019
IFR: infection fatality ratio
IHR: infection hospitalisation ratio
LTLA: lower tier local authority
NHS: National Health Service
REACT-1: REal-time Assessment of Community Transmission-1
RT-PCR: reverse transcription polymerase chain reaction
SARS-CoV-2: Severe Acute Respiratory Syndrome Coronavirus 2

## References

[1] Weekly epidemiological update on COVID-19 - 2021 May 11. [cited 2021 May 17]. Available from: https://www.who.int/publications/m/item/weekly-epidemiological-update-on-covid-19---11-may-2021

[2] Karlinsky A, Kobak D. Tracking excess mortality across countries during the COVID-19 pandemic with the World Mortality Dataset. Elife. 2021:10. doi: 10.7554/eLife.69336 34190045 PMC8331176

[3] Volz E, Mishra S, Chand M, Barrett JC, Johnson R, Geidelberg L, et al. Assessing transmissibility of SARS-CoV-2 lineage B.1.1.7 in England. Nature. 2021. doi: 10.1038/s41586-021-03470-x 33767447

[4] Eales O, Page AJ, Tang SN, Walters CE, Wang H, Haw D, et al. The use of representative community samples to assess SARS-CoV-2 lineage competition: Alpha outcompetes Beta and wild-type in England from January to March 2021. Microb Genom. 2023:9. doi: 10.1099/mgen.0.000887 36745545 PMC9997751

[5] Davies NG, Jarvis CI, CMMID COVID-19 Working Group, Edmunds WJ, Jewell NP, Diaz-Ordaz K, et al. Increased mortality in community-tested cases of SARS-CoV-2 lineage B.1.1.7. Nature. 2021. doi: 10.1038/s41586-021-03426-1 33723411 PMC9170116

[6] Official UK Coronavirus Dashboard. [cited 2022 Oct 20]. Available from: https://coronavirus.data.gov.uk/

[7] Prime Minister’s Office. Prime Minister announces national lockdown. In: GOV.UK [Internet]. 2021 Jan 4 [cited 2021 Apr 22]. Available from: https://www.gov.uk/government/news/prime-minister-announces-national-lockdown

[8] Vasileiou E, Simpson CR, Shi T, Kerr S, Agrawal U, Akbari A, et al. Interim findings from first-dose mass COVID-19 vaccination roll-out and COVID-19 hospital admissions in Scotland: a national prospective cohort study. Lancet. 2021;397:1646–1657. doi: 10.1016/S0140-6736(21)00677-2 33901420 PMC8064669

[9] Harris RJ, Hall JA, Zaidi A, Andrews NJ, Dunbar JK, Dabrera G. Effect of Vaccination on Household Transmission of SARS-CoV-2 in England. N Engl J Med. 2021;385:759–760. doi: 10.1056/NEJMc2107717 34161702 PMC8262621

[10] COVID-19 Response - Spring 2021 (Summary). [cited 2021 May 17]. Available from: https://www.gov.uk/government/publications/covid-19-response-spring-2021/covid-19-response-spring-2021-summary

[11] PHE Genomics Cell, PHE Outbreak Surveillance Team, PHE Epidemiology Cell, PHE Contact Tracing Data Team, PHE Health, Protection Data Science Team, PHE Joint Modelling Team, NHS Test and Trace Joint Biosecurity Centre, Public Health Scotland and EAVE group, Contributions from the Variant Technical Group Members. SARS-CoV-2 variants of concern and variants under investigation in England - Technical briefing 15, 2021 June 11. Available from: https://assets.publishing.service.gov.uk/government/uploads/system/uploads/attachment_data/file/993879/Variants_of_Concern_VOC_Technical_Briefing_15.pdf

[12] Elliott P, Haw D, Wang H, Eales O, Walters CE, Ainslie KEC, et al. Exponential growth, high prevalence of SARS-CoV-2, and vaccine effectiveness associated with the Delta variant. Science. 2021;374:eabl9551. doi: 10.1126/science.abl9551 34726481 PMC10763627

[13] Bernal JL, Andrews N, Gower C, Gallagher E, Simmons R, Thelwall S, et al. Effectiveness of Covid-19 Vaccines against the B.1.617.2 (Delta) Variant. N Engl J Med. 2021. doi: 10.1056/nejmoa2108891 34289274 PMC8314739

[14] Eales O, Wang H, Haw D, Ainslie KEC, Walters CE, Atchison C, et al. Trends in SARS-CoV-2 infection prevalence during England’s roadmap out of lockdown, January to July 2021. PLoS Comput Biol. 2022;18:e1010724. doi: 10.1371/journal.pcbi.1010724 36417468 PMC9728904

[15] Prime Minister’s Office, Street 10 Downing. Prime Minister confirms move to Step 4. In: GOV.UK [Internet]. 2021 Jul 12 [cited 2021 Aug 23]. Available from: https://www.gov.uk/government/news/prime-minister-confirms-move-to-step-4

[16] Eales O, de Oliveira ML, Page AJ, Wang H, Bodinier B, Tang D, et al. Dynamics of competing SARS-CoV-2 variants during the Omicron epidemic in England. Nat Commun. 2022;13:1–11. 35902613 10.1038/s41467-022-32096-4 PMC9330949

[17] Elliott P, Eales O, Steyn N, Tang D, Bodinier B, Wang H, et al. Twin peaks: The Omicron SARS-CoV-2 BA.1 and BA.2 epidemics in England. Science. 2022;376:eabq4411. doi: 10.1126/science.abq4411 35608440 PMC9161371

[18] Khalili M, Karamouzian M, Nasiri N, Javadi S, Mirzazadeh A, Sharifi H. Epidemiological characteristics of COVID-19; A systemic review and meta-analysis. bioRxiv medRxiv. 2020. doi: 10.1101/2020.04.01.20050138 PMC7343974 32594937

[19] Hawryluk I, Mellan TA, Hoeltgebaum H, Mishra S, Schnekenberg RP, Whittaker C, et al. Inference of COVID-19 epidemiological distributions from Brazilian hospital data. J R Soc Interface. 2020;17:20200596. doi: 10.1098/rsif.2020.0596 33234065 PMC7729050

[20] Barnard RC, Davies NG, Jit M, John Edmunds W. Interim roadmap assessment: prior to step 4. [cited 2021 Aug 23]. Available from: https://assets.publishing.service.gov.uk/government/uploads/system/uploads/attachment_data/file/993361/S1290_LSHTM_Roadmap_Step_4.pdf

[21] Sonabend R, Whittles LK, Imai N, Knock ES, Perez-Guzman PN, Rawson T, et al. Evaluating the Roadmap out of Lockdown: modelling step 4 of the roadmap in the context of B.1.617.2. [cited 2021 Aug 23]. Available from: https://assets.publishing.service.gov.uk/government/uploads/system/uploads/attachment_data/file/993427/S1289_Imperial_Roadmap_Step_4.pdf

[22] Keeling MJ, Dyson L, Hill E, Moore S, Tildesley M. Road map scenarios and sensitivity: Step 4. [cited 2021 Aug 23]. Available from: https://assets.publishing.service.gov.uk/government/uploads/system/uploads/attachment_data/file/993358/s1288_Warwick_RoadMap_Step_4.pdf

[23] Knock ES, Whittles LK, Lees JA, Perez-Guzman PN, Verity R, FitzJohn RG, et al. Key epidemiological drivers and impact of interventions in the 2020 SARS-CoV-2 epidemic in England. Sci Transl Med. 2021;13. doi: 10.1126/scitranslmed.abg4262 34158411 PMC8432953

[24] Verity R, Okell LC, Dorigatti I, Winskill P, Whittaker C, Imai N, et al. Estimates of the severity of coronavirus disease 2019: a model-based analysis. Lancet Infect Dis. 2020;20:669–677. doi: 10.1016/S1473-3099(20)30243-7 32240634 PMC7158570

[25] Chemaitelly H, Tang P, Hasan MR, AlMukdad S, Yassine HM, Benslimane FM, et al. Waning of BNT162b2 Vaccine Protection against SARS-CoV-2 Infection in Qatar. N Engl J Med. 2021. doi: 10.1056/NEJMoa2114114 34614327 PMC8522799

[26] Goldberg Y, Mandel M, Bar-On YM, Bodenheimer O, Freedman L, Haas EJ, et al. Waning Immunity after the BNT162b2 Vaccine in Israel. N Engl J Med. 2021;385:e85. doi: 10.1056/NEJMoa2114228 34706170 PMC8609604

[27] Andrews N, Tessier E, Stowe J, Gower C, Kirsebom F, Simmons R, et al. Duration of Protection against Mild and Severe Disease by Covid-19 Vaccines. N Engl J Med. 2022;386:340–350. doi: 10.1056/NEJMoa2115481 35021002 PMC8781262

[28] Bar-On YM, Goldberg Y, Mandel M, Bodenheimer O, Freedman L, Kalkstein N, et al. Protection of BNT162b2 Vaccine Booster against Covid-19 in Israel. N Engl J Med. 2021;385:1393–1400. doi: 10.1056/NEJMoa2114255 34525275 PMC8461568

[29] Riley S, Atchison C, Ashby D, Donnelly CA, Barclay W, Cooke G, et al. REal-time Assessment of Community Transmission (REACT) of SARS-CoV-2 virus: Study protocol. Wellcome Open Res. 2020;5:200. doi: 10.12688/wellcomeopenres.16228.2 33997297 PMC8095190

[30] Riley S, Ainslie KEC, Eales O, Walters CE, Wang H, Atchison C, et al. Resurgence of SARS-CoV-2: detection by community viral surveillance. Science. 2021. doi: 10.1126/science.abf0874 33893241 PMC8158959

[31] Ricoca Peixoto V, Nunes C, Abrantes A. Epidemic Surveillance of Covid-19: Considering Uncertainty and Under-Ascertainment. Port J Public Health. 2020;38:23–29. 10.1159/000507587 PMC7206356 40504207

[32] Li Q, Guan X, Wu P, Wang X, Zhou L, Tong Y, et al. Early Transmission Dynamics in Wuhan, China, of Novel Coronavirus-Infected Pneumonia. N Engl J Med. 2020;382:1199–1207. doi: 10.1056/NEJMoa2001316 31995857 PMC7121484

[33] Zhang G, Hu C, Luo L, Fang F, Chen Y, Li J, et al. Clinical features and short-term outcomes of 221 patients with COVID-19 in Wuhan, China. J Clin Virol. 2020;127:104364. doi: 10.1016/j.jcv.2020.104364 32311650 PMC7194884

[34] Colman E, Puspitarani GA, Enright J, Kao RR. Ascertainment rate of SARS-CoV-2 infections from healthcare and community testing in the UK. J Theor Biol. 2023;558:111333. doi: 10.1016/j.jtbi.2022.111333 36347306 PMC9636607

[35] Pietzonka P, Brorson E, Bankes W, Cates ME, Jack RL, Adhikari R. Bayesian inference across multiple models suggests a strong increase in lethality of COVID-19 in late 2020 in the UK. PLoS ONE. 2021;16:e0258968. doi: 10.1371/journal.pone.0258968 34818345 PMC8612566

[36] Kifer D, Bugada D, Villar-Garcia J, Gudelj I, Menni C, Sudre C, et al. Effects of Environmental Factors on Severity and Mortality of COVID-19. Front Med. 2020;7:607786. doi: 10.3389/fmed.2020.607786 33553204 PMC7855590

[37] Sheikh A, Mcmenamin J, Taylor B, Robertson C, Scotland PH, the EAVE II Collaborators. SARS-CoV-2 Delta VOC in Scotland: demographics, risk of hospital admission, and vaccine effectiveness. Lancet. 2021;397:2461–2462. 34139198 10.1016/S0140-6736(21)01358-1 PMC8201647

[38] Twohig KA, Nyberg T, Zaidi A, Thelwall S, Sinnathamby MA, Aliabadi S, et al. Hospital admission and emergency care attendance risk for SARS-CoV-2 delta (B.1.617.2) compared with alpha (B.1.1.7) variants of concern: a cohort study. Lancet Infect Dis. 2021. doi: 10.1016/S1473-3099(21)00475-8 34461056 PMC8397301

[39] Abu-Raddad LJ, Chemaitelly H, Ayoub HH, AlMukdad S, Yassine HM, Al-Khatib HA, et al. Effect of mRNA Vaccine Boosters against SARS-CoV-2 Omicron Infection in Qatar. N Engl J Med. 2022;386:1804–1816. doi: 10.1056/NEJMoa2200797 35263534 PMC8929389

[40] Jara A, Undurraga EA, Zubizarreta JR, González C, Pizarro A, Acevedo J, et al. Effectiveness of homologous and heterologous booster doses for an inactivated SARS-CoV-2 vaccine: a large-scale prospective cohort study. Lancet Glob Health. 2022;10:e798–e806. doi: 10.1016/S2214-109X(22)00112-7 35472300 PMC9034854

[41] Nyberg T, Ferguson NM, Nash SG, Webster HH, Flaxman S, Andrews N, et al. Comparative analysis of the risks of hospitalisation and death associated with SARS-CoV-2 omicron (B.1.1.529) and delta (B.1.617.2) variants in England: a cohort study. Lancet. 2022;399:1303–1312. doi: 10.1016/S0140-6736(22)00462-7 35305296 PMC8926413

[42] Eales O, Walters CE, Wang H, Haw D, Ainslie KEC, Atchison CJ, et al. Characterising the persistence of RT-PCR positivity and incidence in a community survey of SARS-CoV-2. Wellcome Open Res. 2022;7:102.

[43] Evans C, Barclay W, Zambon M, Horby P, Hiscox J. Dynamics of infectiousness and antibody responses. NERVTAG. Available from: https://assets.publishing.service.gov.uk/government/uploads/system/uploads/attachment_data/file/895788/S0518_NERVTAG_paper_-_viral_dynamics_of_infectiousness.pdf

[44] Park N Population estimates for the UK, England and Wales, Scotland and Northern Ireland - Office for National Statistics. Office for National Statistics; 2020 Jun 23 [cited 2021 Mar 6]. Available from: https://www.ons.gov.uk/peoplepopulationandcommunity/populationandmigration/populationestimates/bulletins/annualmidyearpopulationestimates/latest

[45] COVID-19 Genomic Surveillance – Wellcome Sanger Institute. [cited 2021 Aug 27]. Available from: https://covid19.sanger.ac.uk/lineages/raw?latitude=52.659519&longitude=-1.295191&zoom=5.04

[46] Lang S, Brezger A. Bayesian P-Splines. J Comput Graph Stat. 2004;13:183–212.

[47] Eales O, Ainslie KEC, Walters CE, Wang H, Atchison C, Ashby D, et al. Appropriately smoothing prevalence data to inform estimates of growth rate and reproduction number. Epidemics. 2022;40:100604. doi: 10.1016/j.epidem.2022.100604 35780515 PMC9220254

[48] Hoffman MD, Gelman A. The No-U-Turn Sampler: Adaptively Setting Path Lengths in Hamiltonian Monte Carlo. arXiv [stat.CO]. arXiv; 2011. Available from: http://arxiv.org/abs/1111.4246

[49] Stan Development Team. RStan: the R interface to Stan. 2020. Available from: http://mc-stan.org/

